# Flexible Self-Powered Respiration Sensor Inspired by Fish Lateral Line Systems

**DOI:** 10.34133/research.0905

**Published:** 2025-09-24

**Authors:** Guoliang Ma, Mengze Zhang, Guozhao Shi, Congtian Gu, Hu Shen, Kaixian Ba, Yijie Zhang, Dakai Wang, Xizhao Liu, Bin Yu, Zhiwu Han, Luquan Ren

**Affiliations:** ^1^ State Key Laboratory of Crane Technology, Yanshan University, Qinhuangdao 066000, China.; ^2^ Key Laboratory of Bionic Engineering, Ministry of Education, Jilin University, Changchun 130022, China.; ^3^ Research Institute of Yanshan University, Shenzhen 518063, China.; ^4^ School of Public Administration, Yanshan University, Qinhuangdao 066004, China.

## Abstract

Respiration is a critical physiological signal, and abnormal respiratory patterns are widely recognized as key indicators of various cardiopulmonary and neurological disorders, making continuous monitoring crucial. However, existing clinical systems are constrained by motion limitations, complexity, and cost, hindering daily use. In this work, we present a bio-inspired, low-cost, flexible, and self-powered respiration sensor (BLFS-RS) inspired by the fish lateral line system, operating via contact electrification and electrostatic induction. It features a semicylindrical structure made of silicone rubber, incorporating bionic air channels and a porous-villus composite microstructure. It detects respiratory forces by deforming with abdominal cavity fluctuations, enabling respiration monitoring. Experimental results show that the incorporation of the porous-villus structure increases the sensor’s peak output voltage by 556% compared to a nonstructured counterpart. Additionally, the BLFS-RS exhibits high sensitivity (0.88 V/kPa), high durability (300,000 cycles), and fast response time (48 ms). A wearable real-time respiratory monitoring system based on BLFS-RS enables the acquisition of a comprehensive range of human respiratory parameters, including respiratory rate, apnea–hypopnea index, and peak expiratory flow. Furthermore, patients suffering from speech impairments can utilize the BLFS-RS for alternative communication by encoding information through distinct respiratory patterns. This work is expected to provide a viable and sustainable solution for personalized healthcare and remote respiratory monitoring.

## Introduction

Respiration is a continuous and critical clinical indicator. Abnormalities in respiratory patterns are frequently regarded as early signs of serious diseases such as cardiopulmonary failure [1,2]. Thus, continuous respiratory monitoring is vital for the early detection of human diseases and animal health management. In human health management, respiratory analysis has emerged as a key technology for the early detection of respiratory diseases such as asthma, obstructive sleep apnea, and chronic obstructive pulmonary disease (COPD). By capturing abnormal respiratory rhythms in real time, the risk of acute respiratory failure and sudden cardiac death can be substantially reduced [3,4]. Continuous respiratory monitoring is particularly important for infants and the elderly with neurodegenerative diseases such as Parkinson’s and Alzheimer’s. These systems have demonstrated the ability to provide timely alerts and clinical interventions for sleep-related breathing events, thereby preventing secondary organ damage. Despite its clinical significance, respiratory status remains one of the most challenging vital signs to monitor [5–7]. Currently, respiratory status monitoring often relies on healthcare professionals using specialized equipment to perform measurements. Although accurate, this approach is expensive, complex, and restricts patient mobility, thereby limiting its use to clinical settings. This makes it unsuitable for continuous respiratory monitoring. Intermittent respiratory monitoring, combined with the patient’s awareness of being observed, may result in the loss of critical respiratory data [8,9].

The development of universal wearable bioelectronic systems for continuous respiratory monitoring has the potential to facilitate a paradigm shift toward personalized, preventive, and predictable healthcare. Current wearable respiratory monitoring systems can be divided into 2 main categories based on sensing principles: nasal/oral flow-detecting sensors [10–16] and chest/abdominal volume change sensors [17–22]. Nasal/oral airflow detection sensors monitor respiratory activity by capturing periodic variations in airflow parameters such as temperature and humidity during inhalation and exhalation. Although cost-effective and easy to operate, this sensing approach is highly susceptible to environmental interference. The sensitivity of the sensors experiences a substantial decline when the ambient temperature and humidity reach the parameters of the exhaled gases. Additionally, the temperature sensors are vulnerable to heat accumulation in the absence of effective heat dissipation, resulting in performance degradation and signal drift. Humidity sensors require a well-designed ventilation structure and a hydrophobic protective layer to prevent water vapor condensation. This not only increases the system’s complexity and cost but also affects its reliability over time. Devices worn on the face often induce perceived discomfort and reduce user acceptability by compromising both wearability and comfort. In animal monitoring scenarios, they may even trigger stress responses. Chest/abdominal volume change sensors capture the expansion and contraction of the thorax and abdomen to characterize respiration. Despite the fact that accelerometer-based systems have been demonstrated to be effective in capturing the characteristics of thoracic and abdominal movements, their signal-to-noise ratios are relatively low and they are susceptible to interference from motion artifacts [23]. Recently, textile-based sensors [24–26], electrically conductive polymer composites [27–30], micromachined adhesive patches [31–34], and other flexible strain-sensing technologies have shown promising applications. However, the aforementioned solutions are contingent on external energy supply systems and entail complex preparation processes and customized material formulations, which results in diminished system reliability and escalating manufacturing costs. This, in turn, hinders the clinical translation of wearable respiratory sensors. Therefore, the development of low-cost, flexible, self-powered wearable sensors for continuous respiratory monitoring remains a pressing challenge and a key direction for advancing practical clinical applications.

This paper is inspired by the fish lateral line system and introduces a bio-inspired, low-cost, flexible, and self-powered respiration sensor (BLFS-RS) based on the coupling of contact electrification and electrostatic induction. First, the working mechanism and structural design of the respiratory monitoring device are described. Second, the basic electrical properties and load response of the BLFS-RS are characterized. Third, the sensor’s applicability in various scenarios is demonstrated, including respiratory disease detection, sleep apnea monitoring, and respiration-based human–machine interaction. The conclusion section discusses future directions for the optimization of the BLFS-RS. Materials and Methods, together with the Supplementary Materials, provides comprehensive descriptions of the sensor’s fabrication process, experimental setup, microstructural characterization, structural design, and theoretical background. The flexible, self-powered respiration sensor presents a promising approach in wearable medical electronics and holds substantial potential for health monitoring and human–machine interaction.

## Results

### The structural design and working principle of BLFS-RS

**Fig. 1.**
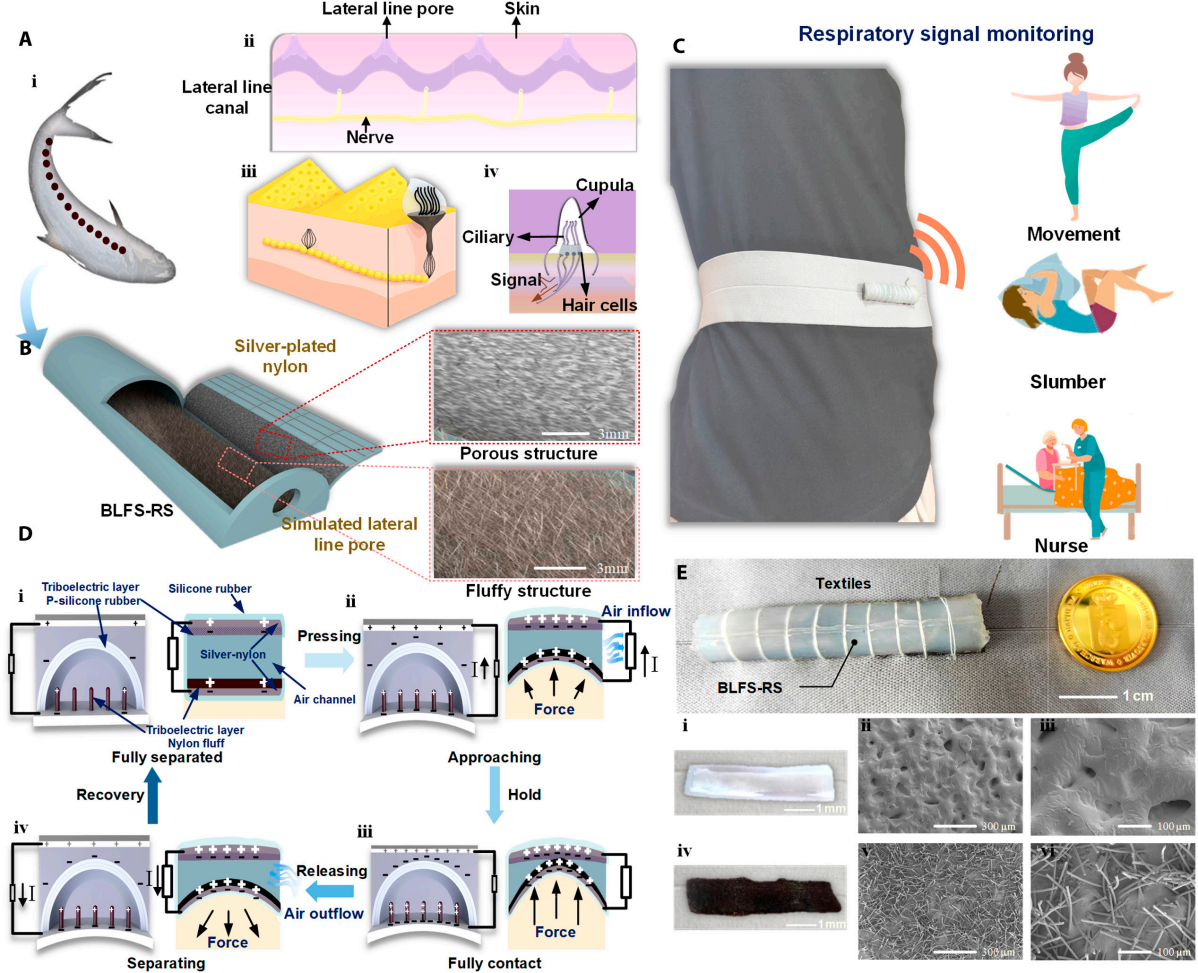
Structure, working principle, and applications of the BLFS-RS. (A) Schematic of the distribution and structure of lateral-line neuromasts. (B) Structural diagram of BLFS-RS. (C) Schematic of BLFS-RS-based respiratory monitoring during movement, slumber, and nurse. (D) Working principle diagram of BLFS-RS. (E) Physical display and SEM images of BLFS-RS.

Serving as a critical environmental sensory organ, the fish lateral line system enables the precise detection of water flow and pressure gradients, enhancing environmental perception [35–37]. It is symmetrically distributed along the body, forming a continuous linear array that extends from the head to the caudal fin (Fig. 1A, i), which increases the fish’s sensitivity to environmental changes. Structurally, the system consists of 2 main components: lateral line neuromasts that detect water flow velocity and lateral line canals that sense pressure variations. A 3-dimensional schematic diagram of the lateral line neuromasts is shown in Fig. 1A, iii. The longitudinal and transverse sections of the lateral line neuromasts are illustrated in Fig. 1A, ii and iv.

The lateral line neuromasts are located within a mucus-filled canal and distributed along the canal wall. The apex of each neuromast is in contact with a structure known as a hair cell, which is enveloped in a gel-like substance. A pressure gradient forms on both sides of the pores due to water flowing through the lateral line pores. This pressure gradient drives mucus movement within the ducts, causing the gel to deflect and activate ion channels in the nerve cells, generating electrical signals. These signals are transmitted via afferent nerve fibers to the fish’s central nervous system, enabling it to perceive and respond to even subtle pressure gradients.

Inspired by this biological mechanism, a BLFS-RS is developed, with its structural design illustrated in Fig. 1B. The sensor is fabricated through a combination of sandpaper molding and electrostatic flocking process, resulting in a semicylindrical silicone rubber structure. This design incorporates internal air channels, a porous matrix, and nylon fluff structures. The gel-covered hair cells in fish neuromasts are mimicked by the sensor’s friction layer, which acts as both a support structure and a triboelectric layer. Lateral through-holes in the silicone rubber simulate the lateral line pores, reducing air entrapment and improving dynamic responsiveness by eliminating internal buffering delays. Silver-plated nylon fibers embedded in the silicone act as conductive pathways, analogous to biological sensory nerves. Details of the fabrication process are provided in the “Fabrication of the flexible self-powered breathing sensor” section. As shown in Fig. 1C, the BLFS-RS can be integrated into wearable textiles positioned on the abdomen to detect deformation caused by respiratory movements, converting mechanical stimuli into continuous analog voltage signals. The high flexibility of the materials ensures excellent conformability to body contours, enabling rapid response to mechanical deformations caused by respiratory activity, and allows continuous monitoring of respiratory information during movement, slumber, and nurse. The BLFS-RS operates based on the coupled mechanisms of the coupling of contact electrification and electrostatic induction [38–43]. To illustrate the working principle more intuitively, the charge transfer processes at both macroscopic and microscopic levels are analyzed (Fig. 1D, i to iv). In the initial state, owing to the difference in electron affinity between the 2 materials, the silicone rubber surface becomes negatively charged and the nylon fibers become positively charged, with no charge flow in the circuit (Fig. 1D, i). Upon application of external pressure, the nylon fibers come into contact with the porous silicone layer, embedding partially into its structure. This contact induces a potential difference between the upper and lower layers, driving electron flow through the silver-coated fibers, thereby generating current (Fig. 1D, ii). As the external force increases, the silicone rubber compresses further, and the charge transfer on the nylon yarn reaches its peak (Fig. 1D, iii).When the external force is released, the nylon fibers retract to their original position, and a reverse current is produced due to the potential difference between the silicone and ground (Fig. 1D, iv). This completes a full energy conversion cycle. The overall length of the RLFS-RS is only 6 cm, and its width is 1 cm, comparable to the size of a coin, as shown in Fig. 1E. Figure 1E, i to iii, present the device and scanning electron microscope (SEM) images of the porous silicone rubber, while Fig. 1E, iv to vi, present the device and SEM images of the nylon fluff.

### The output characteristics research of BLFS-RS

**Fig. 2.**
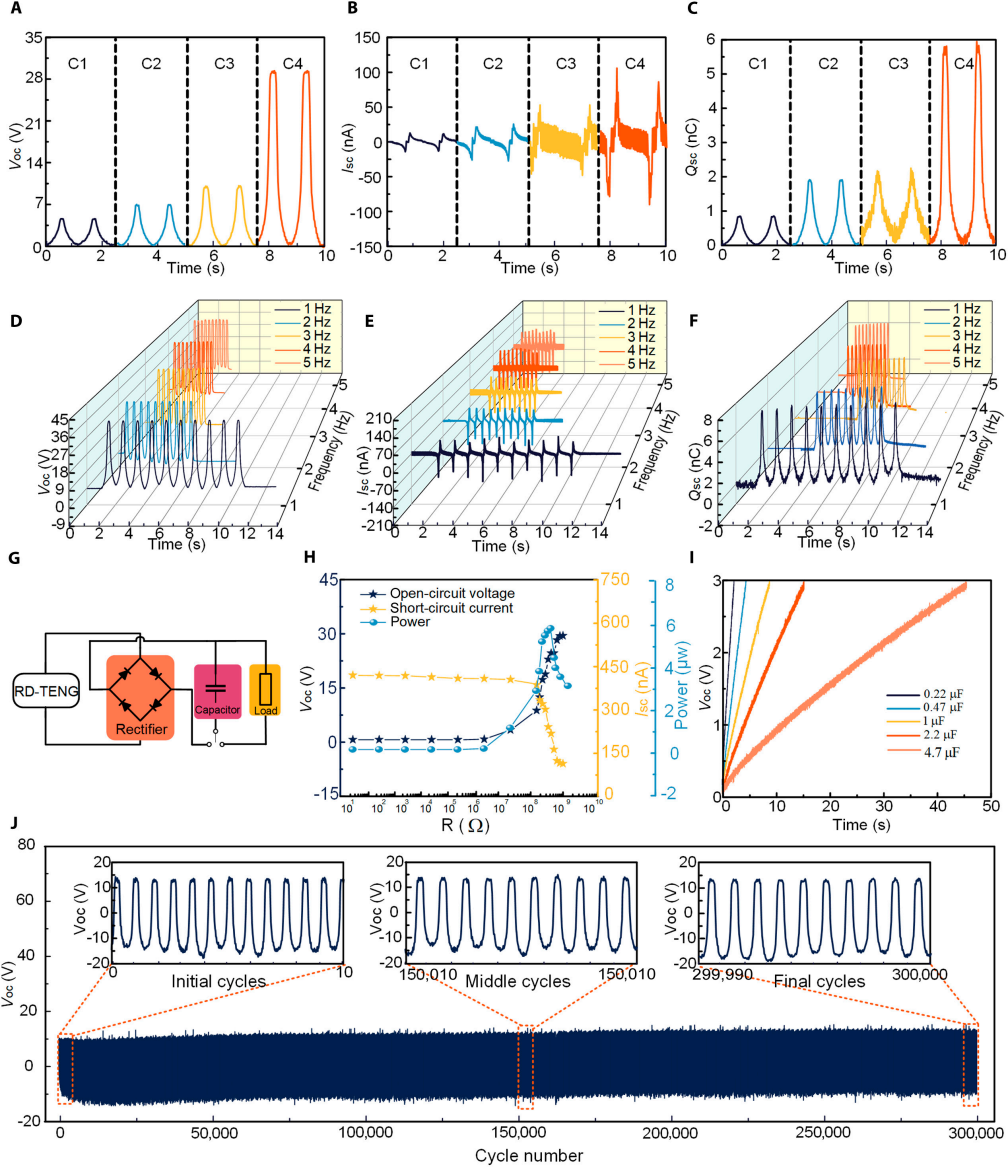
Application performance of BLFS-RS. (A to C) Open-circuit voltage, short-circuit current, and charge transfer of BLFS-RS at different microstructures. (D to F) Open-circuit voltage, short-circuit current, and charge transfer of BLFS-RS at different operating frequencies. (G) Output voltage and current under varying external load resistances. (H) Output power of BLFS-RS under varying external load resistances. (I) Charging curves of capacitors with different capacitances (0.22, 0.47, 1, 2.2, and 4.7 μF). (J) Output voltage of BLFS-RS during 300,000 operating cycles.

A comparative analysis is conducted on the electrical properties of BLFS-RS devices with different cross-sectional geometries (Figs. S1 to S4). The experimental results indicate that the semicircular cross-sectional configuration exhibits superior performance in terms of output voltage, output current, and charge transfer compared to the other 2 geometries. As a result, this configuration is selected as the optimal structural design for BLFS-RS devices. To further illustrate the performance advantages conferred by the biomimetic microstructure of BLFS-RS, sensors with various surface morphologies are fabricated. Subsequently, their electrical performances are evaluated, as shown in Fig. 2A to C, which presents voltage, current, and charge output curves for the different microstructures. Specifically, C1 incorporates a nonporous with no fluff; C2 incorporates a nonporous with fluff; C3 incorporates a porous with no fluff; and C4 incorporates a porous with fluff. Among these, the composite biomimetic structure C4—corresponding to the BLFS-RS design—achieves a maximum output voltage increase of 556% relative to C1, demonstrating the synergistic enhancement effects of the porous and hair-like features.

This substantial performance improvement can be attributed to 3 synergistic mechanisms: (a) The porous structure regulates the effective thickness and dielectric constant of the dielectric layer. Upon application of external force, air is expelled from the porous silicone rubber, substantially reducing the dielectric layer thickness. Simultaneously, the increased material density enhances the dielectric constant, leading to an increase in capacitance and, consequently, a higher charge density on the friction layer. (b) The introduction of a porous surface with fluff substantially increases the effective contact area. Compared to flat configurations, the composite friction layer overcomes the limitations of planar contact, thereby facilitating more efficient charge transfer. (c) The alteration of the friction layer material composition has been shown to optimize the polarity of contact charges and friction performance. Embedding nylon fibers into the silicone rubber surface transforms the friction layer into a composite structure. Given the large triboelectric difference between nylon and silicone, this heterogeneous interface substantially improves charge separation efficiency, enhancing the triboelectric output.

As further demonstrated in Figs. S5 to S7, increasing the applied force from 1 to 9 N results in corresponding output increases: voltage from 9.13 to 36.33 V, current from 49.47 to 199.69 nA, and transferred charge from 1.91 to 9.77 nC. We also evaluated the response time and sensitivity of the BLFS-RS, as shown in Figs. S8 and S9. The BLFS-RS exhibits a response time of 48 ms and a recovery time of 42 ms. The voltage detection area can be divided into 2 distinct regions based on the sensitivity coefficient: a high-sensitivity region below 4.87 kPa, and a low-sensitivity region above 4.87 kPa. The voltage sensitivity coefficients are 0.88 and 0.29 V/kPa, respectively. As demonstrated in Table S1, in comparison with preceding studies, BLFS-RS exhibits substantial advantages in terms of response time, sensitivity, number of cycles permitted, and external power requirements. These enhancements are attributed to compressive deformation of the triboelectric layer, which enlarges the effective contact area and facilitates charge transfer. Additionally, compression of the porous structure reduces the dielectric layer thickness, thereby increasing the equivalent capacitance and surface charge density—ultimately improving the electrical output performance of the BLFS-RS. The output characteristics of the BLFS-RS at varying operational frequencies are illustrated in Fig. 2D to F. Performance tests are carried out at frequencies ranging from 1 to 5 Hz, while maintaining a constant contact force of 9 N. The experimental results indicate that as the frequency increases, the output voltage, current, and transferred charge also increase accordingly. Among the tested frequencies, optimal performance is observed at 5 Hz. Notably, under the optimized conditions of 9 N contact force and 5 Hz frequency, the device delivers peak output values of approximately 36.76 V, 338.16 nA, and 6.98 nC. We analyzed temperature (Fig. S10) and humidity (Fig. S11). The experimental results showed that changes in temperature had little effect on the BLFS-RS sensor. However, as humidity increased, the sensor’s output voltage decreased from 12.3 to 2.05 V.In high-humidity environments, the primary reason for the decrease in output voltage is the ventilation holes added to enhance performance. Increased environmental humidity causes charge leakage due to moisture, resulting in substantial humidity-induced effects on the sensor. To mitigate this, we added a sealing layer to the exterior of the BLFS-RS sensor, comparing the output voltage at 40% humidity with and without the layer. As shown in Figs. S12 and S13, the sensor’s output voltage decreased slightly after sealing, but its waterproof performance improved substantially. Further validation showed that, even after 5 washes in a magnetic stirrer, the sensor’s output voltage remained virtually unchanged, demonstrating that the sealing layer enhances stability and reliability in humid environments. Additionally, we conducted performance tests on BLFS-RS sensors with and without a sealing layer at 80% humidity. As illustrated in Fig. S14, the experimental results suggest that the BLFS-RS sensor with a sealing layer can sustain stable output at 80% humidity, thus avoiding the detrimental effects of high-humidity environments. The equivalent circuit model of the BLFS-RS functioning as a power source for external load resistors is depicted in Fig. 2G. This circuit consists of the BLFS-RS, a rectifier, and a capacitor. The alternating current generated by the device is rectified and stored in the capacitor, providing energy to drive an external load. The electrical output characteristics—voltage, current, and power—under various resistive loads are presented in Fig. 2H. The maximum output voltage and current recorded are 30.53 V and 393.62 nA, respectively. At an external resistance of 180 MΩ, the output power reaches its maximum value of 5.83 μW. As shown in Fig. 2I, capacitors with different capacitances exhibit distinct charging behaviors. The time required to charge commercial capacitors of 0.22, 0.47, 1, 2.2, and 4.7 μF to a voltage of 3 V was measured to be 2.3, 4.31, 8.62, 15.23, and 45.4 s, respectively. Fatigue resistance is a critical performance parameter for wearable electronic devices. To assess this property, 300,000 mechanical loading cycles are performed using a stepper motor operating at 3 Hz under a constant contact pressure of 5 N. The output voltage stability over the test duration is illustrated in Fig. 2J. Remarkably, the BLFS-RS maintained a stable output voltage even after 300,000 cycles, demonstrating its excellent fatigue resistance and long-term operational durability. To investigate the effect of deformation on BLFS-RS performance further, we tested its performance output under different bending radii (see Fig. S15). The results of these experiments indicate that bending does not affect the performance of the BLFS-RS sensor. We also tested the performance of the BLFS-RS sensor after 10,000, 20,000, and 30,000 bending cycles. As illustrated in Fig. S16, even after 30,000 cycles, the BLFS-RS sensor’s output voltage remains stable, suggesting that mechanical deformation has minimal impact on the sensor.

### Respiratory monitoring device for diagnosing the respiratory diseases

**Fig. 3.**
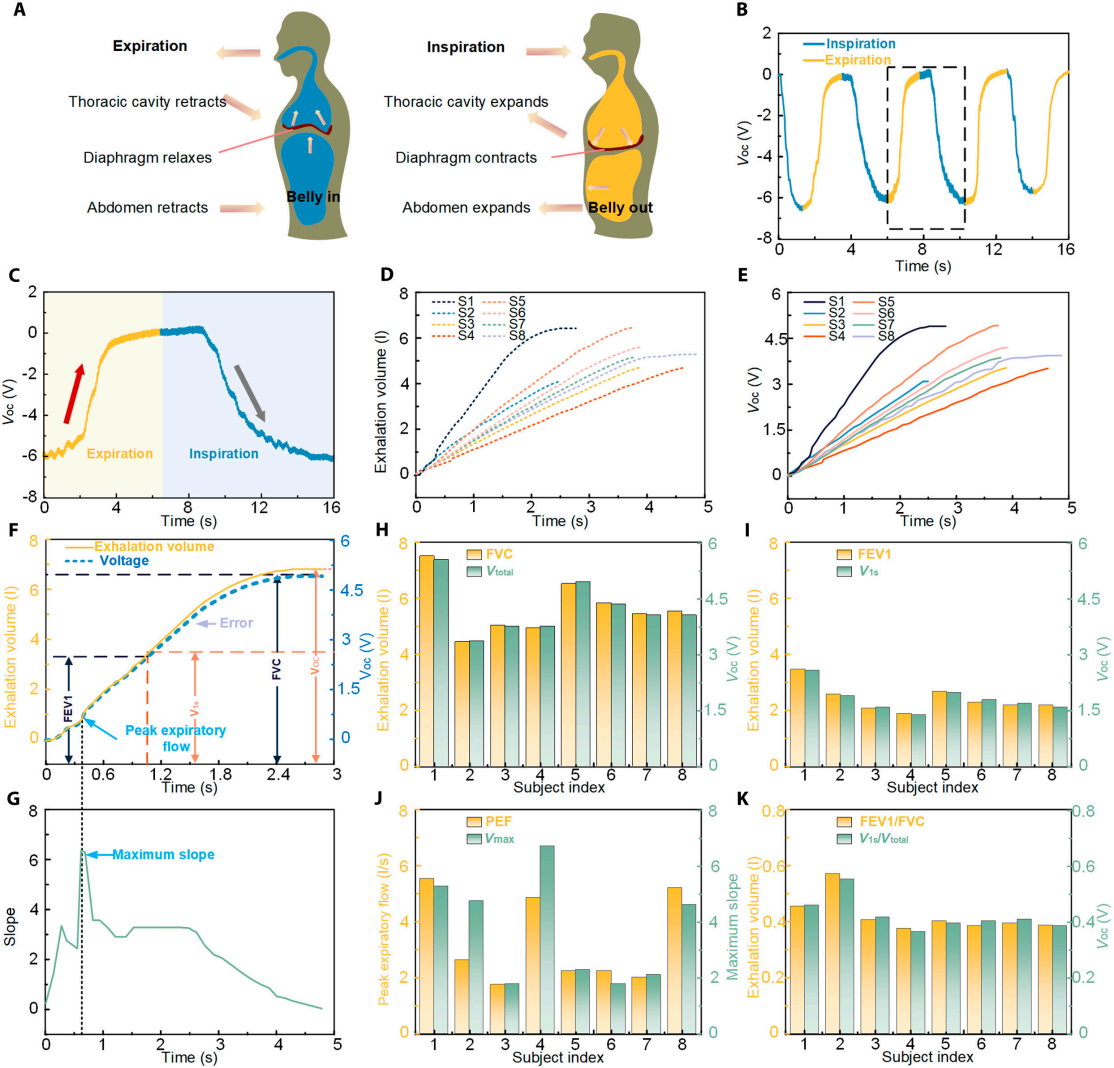
BLFS-RS monitors respiratory system diseases. (A) The mechanism of abdominal dynamic deformation during abdominal breathing. (B) The output voltages of the chest strap based on BLFS-RS. (C) The output voltages of BLFS-RS during a complete respiratory cycle. (D) Curve showing changes in exhaled volume over time measured by a commercial spirometer for 8 subjects. (E) Curve showing changes in electrical signal output from BLFS-RS over time for 8 subjects. (F) Correspondence curve between electrical signals and exhaled air volume over time. (G) Slope curve calculated based on the point signal curve in (F). (H) Comparison chart of FVC and *V*_total_. (I) Comparison chart of FVC_1_ and *V*_1s_. (J) Comparison chart of PEF and *V*_max_. (K) Comparison chart of FEV_1_/FVC and *V*_1s_/*V*_total_.

The process of human respiration is driven by diaphragmatic contraction, which induces the rhythmic expansion and contraction of the abdominal cavity (Fig. 3A). In this experiment, the BLFS-RS is mounted on an elastic band and placed on the abdomen to detect the dynamic deformation of the abdominal circumference during respiration. As the diaphragm descends during inhalation, the abdomen expands, respiring the BLFS-RS and generating a positive electrical signal. During exhalation, the abdomen contracts, releasing the tension on the sensor, which generates a negative electrical signal (Fig. 3B and Fig. S17). For further details on the respiratory signal acquisition process, please refer to Note S1 and the accompanying Figs. S18 to S20. Furthermore, an analysis was conducted on the change curve of the SNR of BLFS-RS under varying pressures, along with an analysis of the SNR for power frequency interference and environmental vibration during the breathing process (Note S2 and Figs. S21 to S24). The findings indicate that incorporating a shielding layer can effectively enhance the SNR against power frequency interference, while environmental vibration shows minimal influence on the SNR. Furthermore, an analysis was conducted to evaluate the impact of various physiological factors, including eating, talking, and sport, on respiratory signals (Figs. S25 to S28). The experimental results showed that eating and talking caused only minor shifts in the BLFS-RS output voltage and did not exhibit periodic or regular patterns. These characteristics allowed clear differentiation of eating and talking from respiratory signals. When respiration and sport activity occurred simultaneously, the signals from the 2 processes were superimposed. Nevertheless, the respiratory signal could still be clearly separated from the motion signal. Figure 3C shows the electrical signal corresponding to a complete respiratory cycle. The terms “inspiratory volume” and “expiratory volume” refer to the amount of air inhaled and exhaled during a single cycle. The exhaled volume is measured using a commercial electronic spirometer (Fig. S29) and the BLFS-RS fixed to the upper abdomen. Figure 3D presents the exhaled air volume measured by the spirometer for 8 subjects, while Fig. 3E shows the curve from the BLFS-RS for the same 8 subjects. Across 8 measurements, the voltage from the BLFS-RS and the exhaled volume from the spirometer showed good agreement. A detailed comparison of the measurement results from both methods is shown in Fig. S30. In respiratory diagnostics, parameters such as forced vital capacity (FVC), forced expiratory volume in 1 s (FEV_1_), and peak expiratory flow (PEF) are critical for assessing lung function, diagnosing respiratory diseases, and evaluating treatment efficacy. FVC is the maximum volume of air exhaled as forcefully as possible after a deep inhalation, and FEV_1_ represents the volume of air exhaled during the first second of exhalation. The FEV_1_/FVC ratio is a key indicator for diagnosing obstructive and restrictive lung diseases [44]. The application of these parameters in disease diagnosis is detailed in Note S3.

These parameters can be measured using a commercial spirometer. The relationship between the open-circuit voltage of the BLFS-RS and exhaled volume, as measured by the spirometer, is shown in Fig. 3F. *V*_oc_ refers to the open-circuit voltage generated by the BLFS-RS during a single exhalation and is positively correlated with FVC. The voltage output at the first second of exhalation (*V*_1s_) corresponds to FEV_1_, while the maximum slope of the voltage curve corresponds to PEF (Fig. 3G). Figure 3H to K compares the measured values of FVC, FEV_1_, PEF, and the FEV_1_/FVC ratio with the corresponding open-circuit voltages of BLFS-RS. A slight deviation between the 2 measurement methods is evident, but the overall agreement suggests that BLFS-RS can be effectively used for wearable respiratory monitoring and the assessment of related respiratory diseases. In order to further assess comfort during respiratory disease monitoring, the performance of the BLFS-RS was tested under different tension levels, as shown in Fig. S31. When the tension reached 0.08 N, the output voltage showed signs of saturation, and the pressure exerted by the elastic band on the abdomen was measured at 2 Pa. This pressure is equivalent to a very light touch or pressure, which is typically not associated with discomfort. Consequently, the pressure from the elastic band is minimal and does not induce a perceptible sensation of compression. Additionally, the BLFS-RS, leather, and tape were affixed to the skin of the arm for 24 h, after which a comparison of skin redness was made, as illustrated in Fig. S32. The experimental results suggest that the BLFS-RS application site exhibited minimal redness, demonstrating its capacity to provide long-term comfort and breathability.

### Respiratory monitoring device for sleep apnea

**Fig. 4.**
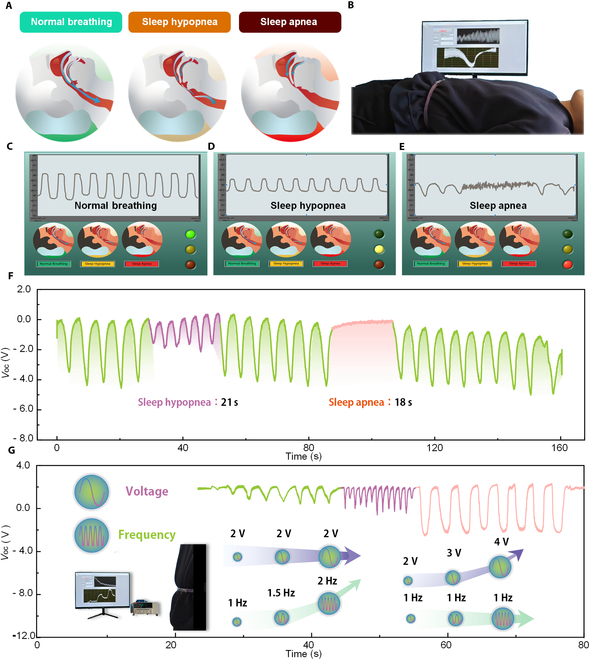
BLFS-RS monitors obstructive sleep apnea–hypopnea syndrome (OSAHS). (A) Breathing status during sleep. The left, middle, and right images represent normal breathing, hypopnea breathing, and sleep apnea, respectively. (B) Photographs of BLFS-RS used for the diagnosis of OSAHS. (C to E) Sleep breathing status judgment panel based on voltage signals. (F) Real-time voltage signals under different sleep breathing statuses. (G) Real-time voltage signals under different breathing patterns.

Obstructive sleep apnea–hypopnea syndrome (OSAHS) is a structural breathing disorder marked by recurrent collapse of the upper airway. The World Health Organization recognizes this condition as one of the most prevalent organic sleep disorders, with a global prevalence ranging from 9% to 38%. In severe cases, complications such as hypertension, arrhythmia, and cognitive impairment may arise [45–48]. Real-time monitoring of respiratory status can help identify sleep apnea symptoms and provide alerts, diagnosis, and appropriate treatment. A wearable real-time respiratory monitoring device based on BLFS-RS, affixed to the abdomen, can continuously detect minor abdominal deformations during sleep. It captures key respiratory waveforms, enabling noninvasive collection and real-time analysis. This enables the development of diagnostic tools for quantifying OSAHS severity and provides preventative measures to mitigate the risk of complications. As shown in Fig. 4A, OSAHS is a respiratory disorder characterized by recurrent airflow reductions or cessations during sleep, caused by pharyngeal narrowing. This leads to gas exchange abnormalities and sleep fragmentation. Obstructive apnea is defined as an interruption of airflow lasting a minimum of 10 s. Similarly, a reduction in thoracoabdominal amplitude of 30% to 50% for at least 10 s is classified as hypoventilation. As shown in Fig. 4B, the diagnostic system for OSAHS includes a volunteer wearing the BLFS-RS on the abdomen, a data acquisition module, and a real-time analysis platform. To measure the respiratory signal characteristics associated with OSAHS symptoms, volunteers control their respiration to simulate hypoventilation and apnea. After the measurement period, the analysis of the 3 typical respiratory states is shown in Fig. 4C to E. The developed LabVIEW-based sleep apnea monitoring system is presented in Fig. S33. If the patient is in a normal respiratory state, the real-time data analysis platform will display a green indicator light. If the patient is in a hypoventilation state for more than 10 s, the real-time data analysis platform will display a yellow indicator light. If the patient remains in apnea for more than 10 s, the platform will display a red indicator light. At the same time, relevant sleep data are automatically saved, including the number of hypopnea and apnea events. These data are used to calculate the apnea–hypopnea index (AHI), a standard metric for assessing the severity of OSAHS. The AHI is quantitatively defined as the total number of apneas and hypopneas recorded per hour of sleep. For further details, refer to Note S4 and Table S2. Figure 4F presents the detailed data from the BLFS-RS. The mean interval and amplitude during normal respiration are 5 s and 3.5 V, respectively. At 30 s, the respiratory amplitude decreased to 1.86 V and 21 s, representing a 47% reduction. At 90 s, a respiratory pause of 18 s was observed, with the electrical signal amplitude dropping to 0.35 V, a 90% decrease. As demonstrated in Movies S1 and S2, BLFS-RS is utilized for the monitoring of sleep respiratory status signals and sleep apnea–hypopnea demonstrations. Figure 4G shows the voltage output signals of BLFS-RS during continuous human respiration monitoring, focusing on 3 different respiratory patterns. Videos depicting continuous respiratory monitoring for the 3 different patterns are provided in Movie S3. A substantial variation in breathing depth and frequency was observed between the 3 breathing modes. The transition from the initial mode to the subsequent mode was characterized by an augmentation in breathing frequency from 1 to 2 Hz, while the breathing depth remained constant. From the first to the third mode, the breathing frequency remained constant, while the output voltage increased from 2 to 4 V as the breathing depth increased.

### Respiratory monitoring device for alternative communication

Respiration is a continuous, spontaneous biomechanical process that occurs throughout the human lifespan. It is largely unconscious and remains stable across various psychological and physiological states [49,50]. For patients with severe spinal cord injuries or disabilities who cannot communicate verbally or move, respiration becomes their only controllable means of interaction. Thus, developing a respiration-driven human–machine interface (HMI) could provide alternative communication between patients and others.

**Fig. 5.**
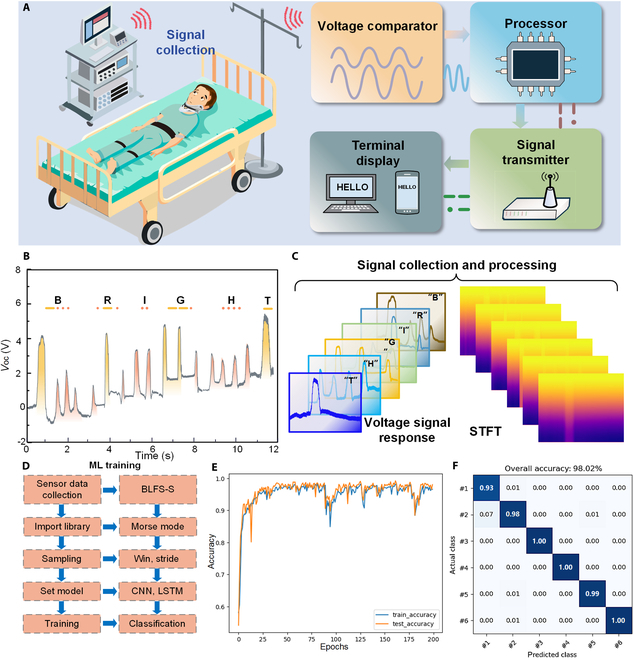
BLFS-RS is used as human–machine interactive interfaces. (A) Schematic diagram of the working principle of BLFS-RS for enabling alternative communication for disabled patients who have lost limb and language abilities. (B) Voltage response curve of Morse code for transmitting the message “BRIGHT” over time. (C) Schematic diagram of voltage signal collection and STFT processing. (D) Schematic diagram of the machine learning training process for assisting Morse code recognition. (E) Training accuracy and testing accuracy. (F) Confusion matrix of voltage outputs for 6 letters.

Morse code, with its simple, efficient, and easily implementable encoding system, has been widely used in various electronic communication scenarios and is highly compatible with binary breathing control patterns (e.g., deep/shallow respiration) [51–53]. We propose the development of a flexible wearable respiratory sensing system based on BLFS-RS. The system transmits information on the user’s state by outputting distinct voltage signals in response to regular respiration, as shown in Fig. 5A. The signal acquisition module collects and compares voltage amplitudes generated by deep and shallow respiration. These signals are mapped to Morse code using a signal processor and encoding algorithm, and then displayed on a terminal for communication and feedback. According to the international Morse code standard (Fig. S34), deep respirations correspond to dashes, while shallow respirations correspond to dots. As shown in Fig. 5B, deep respiration causes greater strain on the BLFS-RS, generating a higher voltage signal. In contrast, shallow respiratory results in less strain and a lower voltage signal. These 2 signals are clearly distinguishable, enabling accurate recognition of both letters and numbers. To improve Morse code recognition from BLFS-RS respiratory signals, the collected voltage signals are processed using short-time Fourier transform (STFT), followed by machine learning (ML) techniques to extract features and classify the collected voltage data (Fig. 5C and Fig. S35). A hybrid convolutional neural network–long short-term memory (CNN-LSTM) model is used to analyze the time-domain characteristics of the voltage signal. As shown in Fig. 5D, the CNN effectively captures local signal features, while the LSTM is adept at modeling temporal dependencies, highlighting voltage differences across time. Figure 5E shows that the training and test accuracies are highly consistent. As shown in Fig. 5F, the model achieved over 93% average accuracy on the test set in classifying 6 Morse code letters. Movie S4 shows the BLFS-RS to be used for respiratory assistance human–computer interface, further highlighting the potential of BLFS-RS for communication support in individuals with disabilities.

## Conclusion

In summary, this paper is inspired by the fish lateral line system and proposes a BLFS-RS, based on contact electrification and electrostatic induction. Experimental results demonstrate that the incorporation of flexible porous silicone rubber as the friction, encapsulation, and support layers, together with nylon fluff and silver-plated nylon yarn as an additional friction layer and as the electrodes, allows the BLFS-RS to maintain stable performance in various application environments. Additionally, the sensor can conform to various curved surfaces and is highly cost-effective, with a material cost of only 1 USD. The BLFS-RS also features high sensitivity (0.88V/kPa), high durability (300,000 cycles), fast response time (48 ms), self-powered capability, and ease of fabrication. The BLFS-RS exhibits a substantial increase in maximum output voltage—up to 6.56 times higher than that of conventional flat structures. This enhancement is attributed to the unique porous fluff composite structure integrated into the design. A wearable real-time respiratory monitoring system based on the BLFS-RS has been developed to monitor multiple human respiratory parameters. Such parameters include respiratory rate, AHI, and PEF. The system supports continuous respiratory monitoring during both physical activity and sleep. For patients with limb paralysis and impaired speech, the BLFS-RS enables communication via respiratory signals. This study offers new insights into continuous respiratory monitoring for both humans, highlighting its potential in health monitoring and smart wearable medical technologies.

Future research will focus on optimizing the interfacial properties of the porous fluff composite structure through multiscale structural engineering, aiming to achieve a pressure resolution in the micropascal (μPa) range. In parallel, the sensor platform will be extended to enable the synchronous acquisition of multimodal physiological signals. In addition, incorporating ML algorithms for dynamic calibration and pattern recognition is expected to improve the sensor’s adaptive performance in real-world environments. Long-term reliability tests under diverse environmental and mechanical conditions will be carried out to support the practical deployment of self-powered biosensing nodes in next-generation medical Internet of Things applications.

## Materials and Methods

### Fabrication of the flexible self-powered breathing sensor

The flexible self-powered respiration sensor, as outlined in this paper, comprises a semicircular porous silicone rubber structure and a square silicone rubber base with nylon fluff affixed to its outer surface. The manufacturing process is detailed in Fig. S36.

#### Fabrication of the semicircular porous silicone rubber

To prepare the silicone rubber solution, the 2 components (Ecoflex00-30, Smooth-On) are mixed in a 1:1 weight ratio. Using a vacuum pump is essential for degassing the mixture, which should be carried out for 5 to 10 min. This step is crucial for eliminating air bubbles. The degassed mixture is cast into 2 types of 3D-printed molds: a semicylindrical mold (60 mm length, 5 mm radius, and 1 mm wall thickness) and a square mold with a sandpaper-textured surface (60 mm × 8 mm × 1 mm, 100 mesh). The molds are allowed to cure at room temperature for 4 h. After demolding, a thin layer of uncured silicone is uniformly applied to the inner surface of the semicylindrical part to serve as an adhesive layer. Silver-plated nylon yarn (0.17 mm in diameter) is arranged in a mesh pattern on the uncured adhesive layer. It is then covered with the square silicone rubber piece from the sandpaper mold, ensuring intimate bonding at the interface. The assembled structure is kept at room temperature for 4 h to ensure complete bonding. The silver-plated nylon yarn embedded between the layers functions as the internal conductive element.

#### Fabrication of the square nylon fluff composite silicone rubber

To prepare the silicone rubber solution, the 2 components (Ecoflex00-30, Smooth-On) are mixed in a 1:1 weight ratio. Using a vacuum pump is essential for degassing the mixture, which should be carried out for 5 to 10 min. This step is crucial for eliminating air bubbles. Next, the silicone rubber solution is injected into 2 square molds (3D-printed, 60 mm long, 10 mm wide, and 1 mm thick) using a syringe. The foundation layer in the electrostatic flocking process is formed by a mold. The electrostatic flocking adhesive (Liwang New Materials Co., Ltd.) is evenly applied to the base silicone rubber layer with a brush. The nylon fibers (diameter 17 μm, 1.5 D × 0.6 mm nozzle) are supplied by Jiahua Craft Printing Materials Co., Ltd., Shaxi Town, Zhongshan City. These fibers are inserted into the electrostatic flocking nozzle. When an electric field is applied, the nylon fibers are propelled and adhere to the base silicone rubber layer. The silicone rubber, with nylon fibers fixed to its surface, is then placed in an oven and baked at 60 to 80 °C for 10 to 20 min. After cooling, a hair dryer is used to remove excess loose fibers. The uncured silicone rubber solution is uniformly applied to the inner surface of a second square silicone rubber sheet to form the adhesive layer. Next, the silver-plated nylon yarn (0.17 mm in diameter) is arranged in a mesh pattern on the uncured silicone rubber solution. The yarn is then covered with a square silicone rubber with nylon fibers fixed to its surface, ensuring a tight seal at the interface. After 4 h at ambient temperature, the electrostatic flocked layer adheres completely to the square silicone rubber, with the silver-plated nylon yarn serving as the internal conductive layer.

An even layer of uncured silicone rubber solution is then applied along the edge of the semicircular porous silicone rubber. The square silicone rubber with nylon fleece is then placed on top of the uncured silicone rubber solution to ensure a tight bond at the interface. The silicone rubber is left at room temperature for 4 h to ensure complete bonding of the upper and lower layers.

### Electrical measurements

The BLFS-RS performance testing platform includes the following components: an electrometer (Keithley 6517), NI data acquisition cards, a computer, a force sensor (HP-50N), and a mechanical motion simulation system driven by a stepper motor (57HB56L4-30DB). The stepper motor-driven linear sliding platform can simulate compression motion, providing energy input to the BLFS-RS. The electrometer (Keithley 6517) and NI data acquisition card are used to measure the open-circuit voltage, short-circuit current, and transferred charge of the BLFS-RS. LabVIEW is used to acquire real-time data, while the force sensor measures the applied force.

## Data Availability

All of the data and figures supporting the findings of this study are provided in the main article or the Supplementary Materials. Additional data concerned with this work are available from K.B. (bkx@ysu.edu.cn), upon reasonable request.

## Supplementary Materials

Supplementary 1 Figs. S1 to S36 Tables S1 and S2 Notes S1 to S4 Movies S1 to S4
