# Supplementary material for: Flexible Self-Powered Respiration Sensor Inspired by Fish Lateral Line Systems
Source: Research (Wash D C). 2025 Sep 24;8:0905. doi: 10.34133/research.0905 (PMC12457744; doi:10.34133/research.0905)
Supplement: Supplementary 1 — Figs. S1 to S36 Tables S1 and S2 Notes S1 to S4 Movies S1 to S4 [file research.0905.f1.zip › supporting information revision.docx]

**SUPPLEMENTARY MATERIALS**

**Flexible self-powered respiration sensor inspired by fish lateral line systems**

*Guoliang Ma^1,2^, Mengze Zhang^1^, Guozhao Shi^1^, Congtian Gu^1^, Hu Shen^1^, Kaixian Ba^1*^, Yijie Zhang^3*^, Dakai Wang^2^, Xizhao Liu^4*^, Bin Yu^1^, Zhiwu Han^2^, Luquan Ren^2^*

1. State Key Laboratory of Crane Technology, Yanshan University, Qinhuangdao 066000, China.

2. Key Laboratory of Bionic Engineering, Ministry of Education, Jilin University, Changchun 130022, China.

3. Research Institute of Yanshan University, R4 Building, Shenzhen Virtual University Park, Nanshan District, Shenzhen 518063, China.

4. School of Public Administration, Yanshan University, Qinhuangdao 066004, China.

* Corresponding author

Email:

bkx@ysu.edu.cn.

zhangyijie@ysu.edu.cn.

liuxizhao@ysu.edu.cn.

**Figure S1.** (a) Photo of BLFS-RS device with semicircular cross-section structure. (b) Photo of BLFS-RS device with square cross-section structure. (c) Photo of BLFS-RS device with circular cross-section structure

**Figure S2.** Output voltage, output current and transferred charge of BLFS-RS with semicircular cross-section structure

**Figure S3.** Output voltage, output current and transferred charge of BLFS-RS with square cross-section structure

**Figure S4.** Output voltage, output current and transferred charge of BLFS-RS with circular cross-section structure

**Figure S5.** Open circuit voltage of BLFS-RS under different load pressures.

**Figure S6.** Short-circuit current of BLFS-RS under different load pressures.

**Figure S7.** Shifting charge of BLFS-RS under different load pressures.

**Figure S8.** Response time of BLFS-RS.

**Figure S9.** Sensitivity of BLFS-RS under different pressure ranges.

**Figure S10.** Effects of different temperatures on the performance of BLFS-RS. (a) Voltage peak variation of BLFS-RS at different temperatures. (b) Open-circuit voltage of BLFS-RS at different temperatures.

**Figure S11.** Effects of different relative humidities on the performance of BLFS-RS. (a) Variation of peak voltage of BLFS-RS under different humidity levels. (b) Open-circuit voltage of BLFS-RS under different humidity levels.

**Figure R12.** Digital photographs of the BLFS-RS cleaning process. (a) Digital photograph of the BLFS-RS after encapsulation. (b) The BLFS-RS was placed in a beaker on a magnetic stirrer and stirred magnetically for 10 minutes at a rotational speed of 800 rpm. After washing and drying, the voltage output characteristics of the BLFS-RS were tested under compression motion (contact pressure of 10 kPa and motion frequency of 1 Hz).

**Figure S13.** Effect of package layer on the voltage output of BLFS-RS and comparison of BLFS-RS with package layer before and after five washings.

**Figure S14.** Comparison of the voltage output of BLFS-RS with and without package layer at 80% humidity.

**Figure S15.** Effects of different bending radius on the performance of BLFS-RS.

**Figure S16.** Effects of different bending cycles on the performance of BLFS-RS.

**Figure S17.** Implementation process of the BLFS-RS for respiratory monitoring.

**Figure S18** Illustration of voltage peak and valley detection in BLFS-RS.


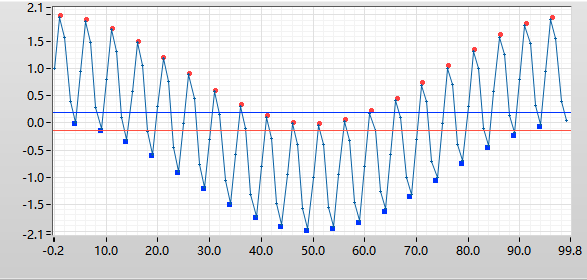


**Figure S19** Real-time peak and valley detection in LabVIEW.

**Figure S20.** Voltage output characteristics of BLFS-RS during expiration and inspiration in a single respiratory cycle. *A_e_* and *A_i_* represent the voltage amplitudes during expiration and inspiration, respectively; *t_e_* and *t_i_* denote the durations of expiration and inspiration. The measured data can be employed to calculate parameters for a range of respiratory modes, for further details.

**Figure S21.** Signal-to-noise ratio (SNR) of the BLFS-RS as a function of pressure.

**Figure S22.** Effect of shield layer on the signal-to-noise ratio (SNR) of BLFS-RS.

**Figure S23.** Effect of environmental vibration frequency on the signal-to-noise ratio (SNR) of BLFS-RS.

**Figure S24.** Effect of environmental vibration amplitude on the signal-to-noise ratio (SNR) of BLFS-RS.

**Figure S25.** Comparison of respiration and eating signals during the use of BLFS-RS as a respiratory monitoring device.

**Figure S26.** Comparison of respiration, talking, and simultaneous respiration and talking signals during the use of BLFS-RS as a respiratory monitoring device.

**Figure S27.** Comparison of respiration, sport, and simultaneous respiration and sport signals during the use of BLFS-RS as a respiratory monitoring device.

**Figure S28.** Effect of physical activity on the signal-to-noise ratio (SNR) of BLFS-RS.

**Figure S29.** The commercial electronic spirometer, which is used to measure the volume of exhaled air

**Figure S30.**The eight measurements measured by a commercial electronic spirometer compare with the corresponding measurements measured by a BLFS-RS.

**Figure S31.** Output voltage of BLFS-RS with different tension forces.

**Figure S32.** Comparison of arm skin condition after 24 hours of fixation with BLFS-RS, leather, and adhesive tape.


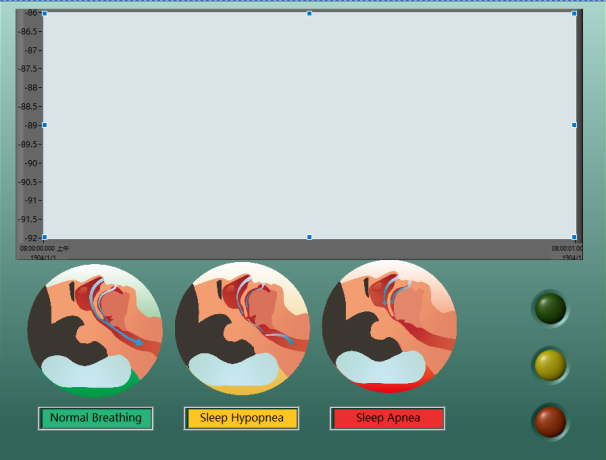

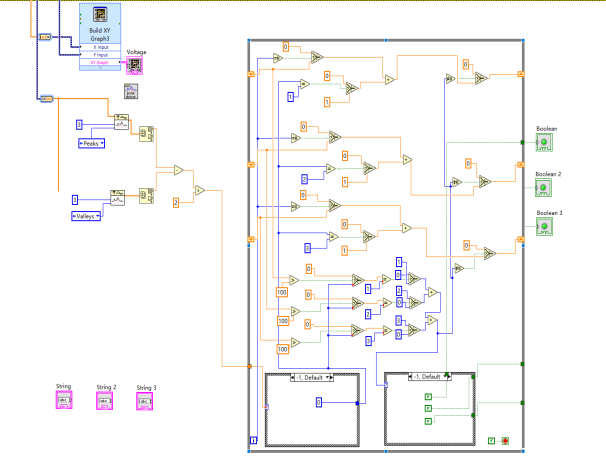


**Figure S33.** The LabVIEW signal acquisition program provides a front panel interface for sleep apnoea monitoring. The graphs are derived from the signals read by the stadiometer and can be recorded and analysed for the severity of OSAHS symptoms.

**Figure S34.** International Morse code encoding.

**Figure S35.** Voltage output signals and corresponding Short-Time Fourier Transform (STFT) for six letters during the use of BLFS-RS as a human-machine interface.

**Figure S36.** The schematic diagram of the BLFS-RS manufacturing method can be found in sections 4.1 and 4.2 of the main text.

**Note S1.** The peak and valley voltage output signals are obtained using the Waveform Peak Detection module in LabVIEW. When applied to the waveform shown in Figure S18, this command enables real-time extraction of peak and valley voltage signals. The real-time peak detection diagram in LabVIEW is shown in Figure S19, where the coordinate points (*t*_max_, *V*_max_) and (*t*_min_, *V*_min_) represent the positions of the waveform peaks and valleys, respectively, as further demonstrated in Figure S20. In the figure, *t_i_* represents the time spent during one inhalation, *t_e_* represents the time spent during one exhalation, *t_i_* and *t_e_* can be used to calculate the respiratory rate. *A_i_* represents the magnitude of the voltage change of BLFS-RS during one inhalation, and *A_e_* represents the magnitude of the voltage of BLFS-RS during one exhalation. *A_i_* and *A_e_* can be used to characterise the depth of respiration, which can be used to monitor the change in respiratory pattern. Therefore, the *i* th *t_i_*(*i*), *t_e_*(*i*), *A_i_*(*i*) and *A_e_*(*i*) can be expressed as follows:

$$t_{i}(i)=t\max(i)-t\min(i)$$

$$t_{e}(i)=t\min(i+1)-t\max(i)$$

$$A_{i}(i)=V\max(i)-V\min(i)$$

$$A_{e}(i)=V\min(i+1)-V\max(i)$$

The time spent during inhalation and exhalation, as well as the magnitude of the voltage change, are known quantities that can be calculated in order to obtain additional information, in addition to the respiratory rate. This additional information can be expressed as the rate of inhalation (*v_i_*), the rate of exhalation (*v_e_*), and the ratio of inhalation to exhalation (*I:E*),and they can be used to judge the existence of abnormal respiratory rhythms, respiratory difficulties and other diseases by observing the changes of *I:E*(*i*), which is important in the field of respiratory system disease monitoring. These values can be expressed for each respiratory movement, with the *i* th value of *v_i_*, *v_e_*, and *I:E* being expressed as follows:

$$v_{i}(i)=\frac{A_{i}(i)}{t_{i}(i)}$$

$$v_{e}(i)=\frac{A_{e}(i)}{t_{e}(i)}$$

$$I:E(i)=\frac{v_{i}(i)}{v_{e}(i)}$$

**Note S2. Calculation of Signal-to-Noise Ratio (SNR)**

The SNR calculation formula is as follows:

$SNR(\text{d}B)=20\lg\frac{ST\text{ouch}}{NT\text{ouch}_{RMS100}}$ (1)

$ST\text{ouch}=S\text{ignal}_{T\text{ouch，}AVG100}-S\text{ignal}_{U\text{ntouch，}AVG100}$ (2)

$Signal_{Touch,avg100}=\frac{\sum_{n=0}^{99} Signal_{Touch}\left[n\right]}{100}$ (3)

$Signal_{Untouch,AVG100}=\frac{\sum_{n=0}^{99} Signal_{Untouch}\left[n\right]}{100}$ (4)

$NTouch_{RMS100}=\sqrt{\frac{\sum_{n=0}^{99} \left(Signal_{Touch}\left[n\right]-Signal_{Touch,AVG100}\right)^{2}}{100}}$ (5)

SNR is a measured amount used in touch sensor controllers that is commonly accepted as an industry-wide standard. Especially in capacitive touch sensors, the signal in SNR is directly related with sensitivity of device performance. The SNR of touch sensor can be modeled using equation (1), where *STouch* is the touch strength from equation (2) and *NTouch* is the standard deviation of the signal in (5). In equation (3) and (4).*Signal_Touch_* is the touch signal level and *Signal_Untouch_* is the untouched signal level.

**Note S3. Clinical Diagnosis of COPD and Asthma: Significance of FVC, FEV1, PEF, and FEV1/FVC**

1. Diagnosis of Chronic Obstructive Pulmonary Disease (COPD):

The diagnosis of Chronic Obstructive Pulmonary Disease (COPD) is made based on the following criteria: In accordance with the diagnostic criteria proposed by the Global Initiative for Chronic Obstructive Lung Disease (GOLD 2023), confirmation of COPD requires the presence of persistent airflow limitation, as demonstrated by pulmonary function tests. This condition is characterized by a post-bronchodilator FEV1/FVC ratio of less than 0.7. Some patients may exhibit respiratory symptoms and/or structural lung damage (e.g., emphysema) and/or physiological abnormalities (including FEV1 at the lower end of normal, gas trapping, lung hyperinflation, impaired lung diffusion capacity, and rapid decline in FEV1), but do not meet the criteria for airflow limitation (i.e., post-bronchodilator FEV1/FVC ≥ 0.7). Individuals exhibiting these characteristics are designated as being in a "pre-COPD state."

2. Diagnosis of Asthma:

According to the diagnostic criteria proposed by the Global Initiative for Asthma (GINA 2023), in a bronchodilator (BD) responsiveness test, adults who, after inhaling a short-acting bronchodilator (e.g., salbutamol 200–400 μg or an equivalent dose) for 10–15 minutes, exhibit an increase in FEV1 of >12% and >200 mL compared to their baseline value, are considered for diagnosis. For children, an increase in FEV1 of >12% compared to their baseline value is required. Additionally, the excessive diurnal peak expiratory flow (PEF) variability test demonstrates that diurnal PEF variability is >10% for adults and >13% for children. These parameter changes are significant criteria for confirming an asthma diagnosis.

**Note S4.**The apnoea hypoventilation index (AHI) serves as a core indicator for assessing the severity of obstructive sleep apnoea hypoventilation syndrome (OSAHS), which is defined as the sum of apnoea and hypoventilation events occurring on average per hour during sleep. According to the OSAHS severity grading criteria listed in Supporting Information Sheet S1, obstructive sleep apnoea events can be clinically staged according to AHI values: mild (5-15 events/hour), moderate (15-30 events/hour) and severe (>30 events/hour).

**Table S1 Properties comparison of the reported mainstream flexible respiratory sensors and that in this work.**

| **Work mode** | **Sensitivity** | **Self-powered** | **Durability** | **Response time** | **Reference** |
| --- | --- | --- | --- | --- | --- |
| TENG | 0.19 V/kPa | Yes | 4500 | 16 ms | Ref1 |
| Piezoresistive | 575542 (GF) | No | 9000 | 100 ms | Ref2 |
| Piezoelectric | 3.33 V/kPa  591 pC/kPa | Yes | 1100000 | 1.67 ms | Ref3 |
| Piezoresistive | 87 (GF) | No | 6500 | — | Ref4 |
| Piezoelectric | 0.19 V/Pa | Yes | 6 hour | — | Ref5 |
| Piezoresistive | 51.53/kPa | No | 8000 | — | Ref6 |
| TENG | 1.33 V/kPa | Yes | 4200 / 4 hour | — | Ref7 |
| TENG | 0.43 V/N | Yes | 10000 | 1 ms | Ref8 |
| TENG | 0.46 V/kPa | Yes | 10000 | 280 ms | Ref9 |
| Strain | — | Yes | 20000 | 70 ms | Ref10 |
| Piezoresistive | 11.8 (GF) | No | 1000 | 94 ms | Ref11 |
| R-C dual-mode | 0.053/kPa | No | 14 days | 20 ms | Ref12 |
| TENG | 0.15 mV/Pa | Yes | 4800 | 4 ms | Ref13 |
| Piezoelectric | 1.8 V/kPa  21 nA/kPa | Yes | 12000 | 40 ms | Ref14 |
| Piezoelectric | — | Yes | 8000 | 50 ms | Ref15 |
| Piezoelectric | 15.5 mV/kPa | Yes | — | 35 ms | Ref16 |
| Strain | 12 | No | 4500 | 16 ms | Ref17 |
| Strain | 10000 | No | 2000 | 600 ms | Ref18 |
| Strain | 9.84–30.8 | No | 100 | — | Ref19 |
| Strain | 1000-2500 | No | 500 | 300 ms | Ref20 |
| **TENG** | **0.88 V/Kpa** | **Yes** | **300000** | **48ms** | **our work** |

**References**

(1) C. Yan, W. Deng, L. Jin, T. Yang, Z. Wang, X. Chu, H. Su, J. Chen, W. Yang, Epidermis-inspired ultrathin 3d cellular sensor array for self-powered biomedical monitoring. ACS Appl. Mater. Interfaces. 10 (2018) 41070-41075.

(2) G.B. Pradhan, K. Shrestha, M. Assaduzzaman, S. Sapkota, S. Lim, M.S. Reza, J.Y. Park, A hybrid porous carbon-decorated multi-layered graphene-based breathable and ultra-sensitive piezoresistive strain sensor for wearable physiological signal monitoring. Chem. Eng. J. 504 (2025) 158933.

(3) L. Han, W. Liang, Q. Xie, J. Zhao, Y. Dong, X. Wang, L. Lin, Health monitoring via heart, breath, and korotkoff sounds by wearable piezoelectret patches. Adv. Sci. 10 (2023) 2301180.

(4) A. Kumar, R.K.R. Kumar, M.O, Shaikh, C.H. Lu, J.Y. Yang, H.L. Chang, C.H. Chuang, Ultrasensitive strain sensor utilizing a agf-agnw hybrid nanocomposite for breath monitoring and pulmonary function analysis. ACS Appl. Mater. Interfaces. 14 (2022) 55402-55413.

(5) J. Zhong, Z. Li, M. Takakuwa, D. Inoue, D. Hashizume, Z. Jiang, Y. Shi, L. Ou, M.O.G. Nayeem, S. Umezu, et al. Smart face mask based on an ultrathin pressure sensor for wireless monitoring of breath conditions. Adv. Mater. 34 (2022) 2107758.

(6) Y. He, L. Zhao, J. Zhang, L. Liu, H. Liu, L. Liu, A breathable, sensitive and wearable piezoresistive sensor based on hierarchical micro-porous pu@cnt films for long-term health monitoring. Compos. Sci. Technol. 200 (2020) 108419.

(7) M. Lou, I. Abdalla, M. Zhu, X. Wei, J. Yu, Z. Li, B. Ding, Highly wearable, breathable, and washable sensing textile for human motion and pulse monitoring. ACS Appl. Mater. Interfaces. 12 (2020) 19965-19973.

(8) S. Wang, M. Tian, S. Hu, W. Zhai, G. Zheng, C. Liu, C. Shen, K. Dai, Hierarchical nanofibrous mat via water-assisted electrospinning for self-powered ultrasensitive vibration sensors. Nano Energy. 97 (2022) 107149.

(9) Y. Fang, J. Xu, X. Xiao, Y. Zou, X. Zhao, Y. Zhou, J. Chen, A deep-learning-assisted on-mask sensor network for adaptive respiratory monitoring. Adv. Mater. 34 (2022) 2200252.

(10) C. Ning, R. Cheng, Y. Jiang, F. Sheng, J. Yi, S. Shen, Y. Zhang, X. Peng, K. Dong, Z.L. Wang, Helical fiber strain sensors based on triboelectric nanogenerators for self-powered human respiratory monitoring. ACS Nano. 16 (2022) 2811-2821.

(11) Y. Cai, H. Song, H. Ni, Y. Wang, M. Dai, J. Lu, Z. Guo, K. Shi, C. Liu, Y. Qing, Amphibious-adaptive superhydrophobic fabric with bi-conductive coupling for robust human motion detection. Chem. Eng. J. 511 (2025) 161800.

(12) J. Liu, H. Wang, T. Liu, Q. Wu, Y. Ding, R. Ou, C. Guo, Z. Liu, Q. Wang, Multimodal hydrogel-based respiratory monitoring system for diagnosing obstructive sleep apnea syndrome. Adv. Funct. Mater. 32 (2022) 2204686.

(13) X. Wang, J. Yang, K. Meng, Q. He, G. Zhang, Z. Zhou, X. Tan, Z. Feng, C. Sun, J. Yang, et al. Enabling the unconstrained epidermal pulse wave monitoring via finger-touching. Adv. Funct. Mater. 31 (2021) 2102378.

(14) Q. Xu, M. Jia, P. Zhou, Y. Zhang, W. Guo, S. Zhao, H. Zeng, J. Zhang, M. Yan, S. Jiang, et al. High-performance ultrasensitive flexible piezoelectric thin film sensors via a cost-effective transfer strategy. Adv. Funct. Mater. 35 (2025) 2414211.

(15) C. Kong, R. Ma, X. Guo, L. Zhang, C. Song, M. Zhang, J. Hu, A non-implantable flexible stretchable sensor for detecting respiratory rhythms in animals. Comput. Electron. Agr. 224 (2024) 109183.

(16) M.A. Signore, G. Rescio, L. Francioso, F. Casino, A. Leone, Aluminum nitride thin film piezoelectric pressure sensor for respiratory rate detection. Sensors. 24 (2024) 2071.

(17) T. Gong, J.X. Guo, H.Q. Shao, J. Jia, K. Ke, R.Y. Bao, W. Yang, Linear strain sensors via a spatial heteromodulus tricontinuous structure design for high-resolution recording of snoring breath. acs appl. mater. interfaces. 15 (2023) 56337-56346.

(18) A. del Bosque, X.X. Fernández Sánchez-Romate, Á. De La Llana Calv, P.R. Fernández, S. Borromeo, M. Sánchez, A. Ureña, Highly flexible strain sensors based on cnt-reinforced ecoflex silicone rubber for wireless facemask breathing monitoring via bluetooth. ACS Appl. Polym. Mater. 5 (2023) 8589-8599.

(19) R.G. Ferreira, A.P. Silva, J. Nunes-Pereira, Conductive and elastomeric rgo@pdms piezoresistive composite for flexible strain sensing applications: joint motion and breathing pattern monitoring. ACS Appl. Electron. Mater. 7 (2025) 6366-6383.

(20) A. del Bosque, X.F. Sánchez–Romate, D. Patrizi, J.S. del Río Sáez, D.Y. Wang, M. Sánchez, A. Ureña, Ultrasensitive flexible strain sensors based on graphene nanoplatelets doped poly(ethylene glycol) diglycidyl ether: mask breathing monitoring for the internet of things. sens. Actuators A Phys. 358 (2023) 114448.

**Table S2 Severity criteria for OSAHS**

| Degree | AHI(times/hour) |
| --- | --- |
| Normal | <5 |
| Mild | 5~15 |
| Moderate | 15~30 |
| Severe | >30 |
